# Effect of behavioral activation on time and frequency domain heart rate variability in older adults with subthreshold depression: a cluster randomized controlled trial in Thailand

**DOI:** 10.1186/s12888-022-03962-8

**Published:** 2022-05-05

**Authors:** Wanvisa Saisanan Na Ayudhaya, Nuttorn Pityaratstian, Wichai Eungpinichpong, Thanapoom Rattananupong, Parinya Kitidumrongsuk, Sriprapa Loonlawong, Wiroj Jiamjarasrangsi

**Affiliations:** 1 Department of Community Public Health, School of Public Health, Walailak University, Nakhon Si Thammarat, Thailand; 2 Research Center of Workers Health, Walailak University, Nakhon Si Thammarat, Thailand; 3 Department of Psychiatry, Faculty of Medicine, Chulalongkorn University, Bangkok, Thailand; 4 Research Center in Back, Neck, and Other Joint Pain and Human Performance, Faculty of Associated Medical Sciences, Khon Kaen University, Khon Kaen, Thailand; 5 Department of Preventive and Social Medicine, Faculty of Medicine, Chulalongkorn University, Bangkok, Thailand; 6 Regional Health Promotion Center 9 Nakhon Ratchasima, Department of Health, Nakhon Ratchasima, Thailand

**Keywords:** Behavioral Activation, Time and Frequency Domain, Heart Rate Variability, Older Adults, Subthreshold Depression, Cluster randomized controlled trial

## Abstract

**Background:**

Increased prevalence of depression highlights the need for effective interventions. Behavioral activation (BA), which can easily be adapted for non-clinical populations, has been the recommended treatment for depression. It is based on a model of psychopathology explaining that losses or chronically low levels of positive reinforcement yield behavioral and emotional changes in depression and that encouraging individuals to increase their engagement in reinforcing activities can improve their mood and enhance their valuable life experiences. Heart rate variability (HRV) provides indices of autonomic function related to depression, but only a few studies have investigated the effect of BA on HRV, particularly among older adults with subthreshold depression. Accordingly, we aimed to investigate the effect of BA on HRV in older adults with subthreshold depression.

**Methods:**

We conducted a 9-month cluster randomized controlled trial in two Health Promoting Hospitals (HPHs). Eighty-two participants were randomized into either intervention (BA with usual care) or control (usual care only) groups, with 41 participants per group. Daily step count was collected weekly during the 12-week BA intervention period, while HRV parameters, including the Standard Deviation of the Normal-to-Normal interval (SDNN), High Frequency (lnHF), Low Frequency (LF), and Low Frequency/High Frequency ratio (LF/HF), were examined at 0, 3, 6 and 9 months. Generalized Estimating Equations (GEEs) were used in the data analysis.

**Results:**

Over nine months, the intervention and control groups differed significantly in the unadjusted mean change of HRV, SDNN [7.59 ms (95% CI: 1.67, 13.50)], lnHF [0.44 ms^2^ (95% CI: 0.04, 0.85)], and LF [0.53 ms^2^ (95% CI: 0.09, 0.98)], whereas the groups did not differ significantly in LF/HF ratio [0.01 ms^2^ (95% CI: -0.04, 0.06)].

**Conclusion:**

Our results suggest that BA may have a therapeutic effect on depression symptoms of older adults with subthreshold depression via improved HRV.

**Trial registration:**

TCTR20211019003, thaiclinicaltrials.org, retrospectively registered on 19 October 2021.

**Supplementary Information:**

The online version contains supplementary material available at 10.1186/s12888-022-03962-8.

## Background

Depression in the geriatric community has been identified as a major problem given its negative outcomes that include poor functioning, increased perception of ill health, and increased utilization of medical services. [1, 2] Almost 14% of individuals aged over 55 exhibit a depressive syndrome, of which only 2% are diagnosed with major depressive disorder (MDD) [3]. Conversely, some older adults affected by depression do not meet the MDD criteria, having a subsyndromal or subthreshold depression (SD). Subthreshold depression is diagnosed when a core symptom is accompanied by an additional one to three depression symptoms and is of clinical significance due to the associated impairment in social and occupational functioning similar to that of major depression [4]. The prevalence of subthreshold depression in older adults is reportedly higher than that of major depression, with international data reporting the prevalence rates of 5 to 37% for SD [5] and 5 to 10% for clinically significant depression [6].

Behavioral Activation (BA), formerly a component of Cognitive Behavior Therapy (CBT), is an alternative therapy for treating older adults with depression that is convenient and cost-effective, showing the same effectiveness as CBT [7]. BA is based on the theory that individuals with depression tend to engage in avoidance and isolation that maintain or worsen their symptoms [8]. Therefore, the treatment goal is to encourage individuals to gradually decrease their avoidance and isolation and increase their engagement in activities that have been shown to improve mood and enhance valuable life experiences [9] In contrast to CBT, BA therapy is brief and simple, and because a non-specialist can administer it with minimal formal training, it is commonly used in primary care services [10, 11]. However, while previous studies have supported the effectiveness of BA for both major depression and sub-threshold depression, they have largely utilized subjective measures, such as patients’ self-assessment, with limited evidence-based or objective measures [12].

One of the underlying pathophysiological mechanisms characterizing depression is autonomic dysfunction that includes decreased parasympathetic and/ or increased sympathetic tone [13, 14]. Heart rate variability (HRV), a non-invasive biomarker of autonomic nervous system response, might therefore represent a useful endophenotype for emotional dysregulation and should be used as an objective measure of the efficacy of prevention/intervention therapies in depression [15]. HRV refers to the beat-to-beat alterations in heart rate, which can be obtained from electrocardiogram (ECG) recordings, and provides information about the dynamic changes in sympathovagal modulation at the cardiac sinoatrial node. HRV can be quantified into either time-domain or frequency-domain. Time-domain indices measure the HRV during monitoring periods, ranging from 2 min to 24 h [16]. The interval between normal QRS complexes or the immediate HR at a specific point is measured to estimate a normal-to-normal interval (NN interval) between consecutive normal QRS complexes. The standard deviation of the NN interval (SDNN) can be determined using the NN interval. SDNN reflects all the cycle components contributing to HRV. The greater the SDNN, the higher the variability of the heart rates transmitted through the parasympathetic nerve. A decrease in SDNN implies reduced coping with stress and the impairment of overall health status and ANS control capacity [17]. Frequency domain analysis of HRV including high frequency (lnHF) and low frequency (LF) spectral components calculated through short-term measurements (i.e., 2–5 min) [18], can reflect the function of the parasympathetic nervous system and sympathetic branches [17]. Reduced InHF variability (parasympathetic modulation and increased LF/HF ratio (suggesting a sympathetic prevalence) have been reported in patients with major depressive disorder [19, 20]. On the contrary, increased resting HRV was associated with positive aspects of psychological makeup such as more adaptive self-regulation and social engagement [21].

While the effectiveness of CBT in treating depressive patients and HRV improvement has been supported [22, 23], no such evidence exists for BA, although BA is assumed to have a beneficial effect on autonomic system regulation the same way as CBT [24]. This study aimed to evaluate the effectiveness of the adapted BA among Thai older adults with sub-threshold depression residing in the community.Program effectiveness was assessed using two objective measures, (a) daily step count as the indicator of daily (physical) activity level and (b) HRV indexes as the biomarkers of depressive symptoms. The results of the program's effectiveness on psychological parameters had been reported previously in a separate article [25].

## Methods

### Participants

**Fig. 1:**
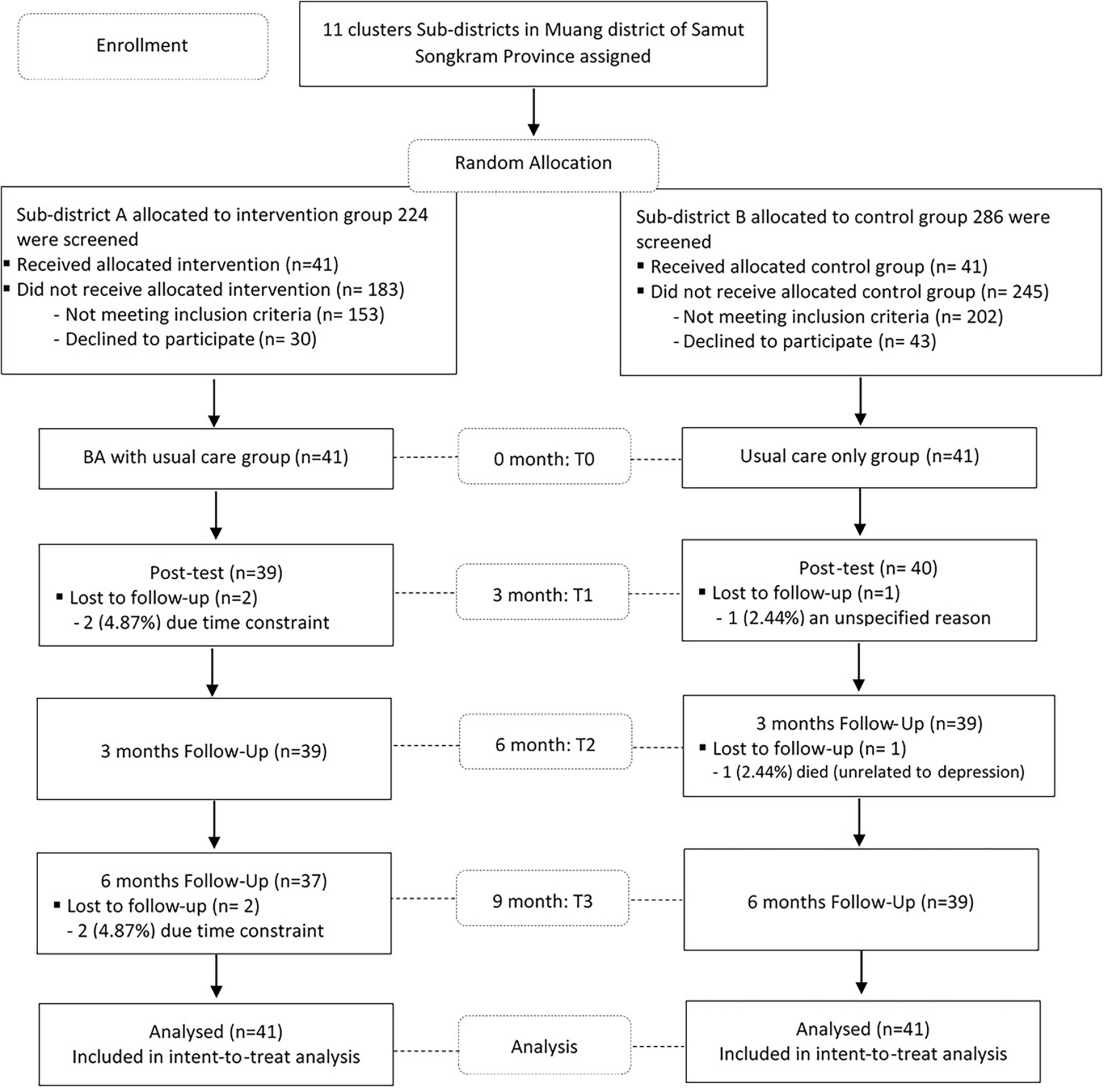
Flowchart of include study participants. BA, behavioral activation; T0, Basline; T1, at the end of BA intervention of the experimental group or at the end of the 3rd month of the study; T2, 3 months follow-up after the end of the intervention or at the end of the 6th month of the study; T3, 6 months follow-up after the end of the intervention or at the end of the 9th month of the study

A single-blind 2-clustered randomized control trial (RCT) was conducted after obtaining approval from the Institutional Review Board of Faculty of Medicine, Chulalongkorn University (IRB No. 680/61). The trial was first registered at the thaiclinicaltrials.org Protocol Registration System (TCTR), retrospectively registered on 19 October 2021with the Registration No. TCTR20211019003. All participants were provided all essential information regarding the study protocols before distributing informed consent. The study was conducted in accordance with the Declaration of Helsinki. The detailed method was described previously [25]. Potential participants were recruited from 2 Health Promoting Hospitals (HPHs) in 2 randomly selected sub-districts in Muang Samut Songkram district of Samut Songkram Province, Thailand (Sub-districts A and B, respectively). Out of 286 and 224 adults aged 60 years or older recruited from these two sub-districts, respectively, 71 and 84 with subthreshold depression (with the Thai Geriatric Depression Scale or TGDS of 13–24; mild to moderate) were identified with the assistance of the officers of the local sub-district Health Promoting Hospitals and local VHVs. Those who met the following criteria were excluded: (1) hearing impairment and/or dementia assessed by the Mini-Mental State Examination Thai version (MMSE-Thai 2002) [26], (2) potentially life-threatening psychiatric and medical comorbidities or conditions that would limit study participation or adherence, and (3) currently undergoing any psychotherapy or taking antidepressants. The remaining 41 participants in sub-districts A and B were included as the BA plus usual care and usual care only groups in the study (Fig. 1).

### Intervention

This BA intervention contained 12 2-h weekly sessions composed of three main steps, activity monitoring, activity scheduling, and modification [27]. It aimed to (a) increase engagement in rewarding activities, (b) decrease avoidance and isolation that maintain depression or increase the risk of depression, and (c) target factors that restrict access to reinforcement or maintain aversive control [27]. The detailed method was described previously [25]. Briefly, WS (Principal investigator), who was a non-mental health professional but had attended a 6-day formal training course in cognitive behavioral therapy (CBT) and BA, administered the BA intervention while a nurse working at the local Health Promoting Hospital and nine local VHVs who would serve as research assistants were recruited and provided 1-day training to research assistants on data collection and management of the participants during the BA intervention period. Groups of 3 VHVs were assigned to conduct follow-ups with and assist 13–14 participants in the residential community.

The first week of BA intervention started with the instruction about the principles and process of BA, the depressive cycle and depression prevention concepts, the activity scheduling introduction, and the recording of the scheduling in forms. The participants were asked to practice activity schedule recording as homework. Participants were encouraged to seek support from family members and VHVs to finish the recording. Later, weekly BA sessions emphasized specific tasks, (1) activity monitoring to examine the effect of specific activities affected mood, (2) activity scheduling to develop a plan to increase pleasant activities, and (3) modification: utilizing problem-solving to alter contextual problems that may be stimulating or maintaining depressed mood: via problem-solving.

To minimize the potential for the Hawthorne effect [28, 29], participants in the usual care group also participated in the psychoeducation in the first session of Week 0 (similar to the intervention group) and then the 12 weekly follow-up sessions. Rather than receiving homework assignment or activity monitoring in each session as in the intervention group, participants in the usual care group underwent regular physical examinations to review their current health symptoms and assess their individual health needs delivered by the local mental health nurse every week for twelve weeks. The investigator (WS) standardized the activities to ascertain the protocol fidelity between the intervention and control groups [25]. GP or primary care mental health worker followed the participants and offered interventions deemed appropriate for their condition according to normal practice.

### Data collection

Demographic data were collected using a baseline questionnaire (M0) assessing age, gender, marital status, educational and income levels, employment, household living statuses, and personal disease history. Baseline levels of depression (measured by TGDS or 30 self-rated items of the Thai geriatric depression scale) [30] and anxiety and stress (measured by DASS questionnaire or 21 items of the self-reported Depression Anxiety Stress Scales) [31] were also assessed to check for baseline comparability between the intervention and control groups in mental health status.

Daily step count data were collected from both groups, BA with usual care and usual care only group, every other week (W0 to W12) for 12 weeks using a pedometer (HJ-325, Omron Health Care Corporation, Kyoto, Japan). Participants were instructed to wear a pedometer on the neck all day during the study except when sleeping, swimming, or bathing. No particular instructions were given about timing walking timing, and participants could walk according to their lifestyle. The pedometer automatically recorded the number of steps taken (steps/day) for up to 6 months, enabling participants to see their “steps/day” data recorded for the seven days before a given assessment day.

HRV data were collected at baseline (M0), 3 months (M3), 6 months (M6), and 9 months (M9) by uBioMacpa (Certificate by KFDA; BioSensecreative Co. Ltd., Seoul, Korea) and its embedded software [32, 33]. Before the HRV test, participants were asked to refrain from smoking or consuming alcohol, coffee, and energy drinks for at least 8 h but were allowed to drink some water. HRV was tested at 8.30 am. After sitting at rest on a comfortable chair for 5 min, a continuous HRV of 2.5 min (ultra-short-term) was recorded. Variables such as talking, coughing, deep breathing, and body movements were controlled [24, 34]. The HRV analysis was performed in the time and frequency domain according to the methodological standards [18]. Multiple data of HRV were calculated as follows. The time-domain included the standard deviation of normal-to-normal intervals (SDNN). The frequency domain (power spectral density; PSD) included low frequency (LF; 0.04–0.15 Hz), high frequency (lnHF; 0.15–0.40 Hz), and the ratio of LF/HF.

### Statistical analysis

Baseline continuous variables were described as the means (standard deviation or SD) under a normal distribution (age, MMSE, TGDS, DASS) and median (interquartile range or IQR) under an asymmetrical distribution (Income). Categorical variables (gender, marital status, education, employment, living status, personal disease history) were described as frequencies (percent).

The within-group comparison of the daily step counts between baseline and subsequent measurements (W0 versus W1-W6) and the between-group comparison were conducted using generalized estimating equations (GEEs). To account for missing data, when the participants forgot to wear their pedometer, we used only data obtained when the length of wearing time exceeded 12 h a day.

The data from the HRV tests were normally distributed and summarized using the means (SD). To evaluate the effect of the BA intervention program on HRV at the group level, GEEs were used to evaluate between-group differences across different time points based on an intention-to-treat (ITT) analysis. This analysis accounted for the potential confounding effect of employment status and education level. P-value of < 0.05 was considered statistically significant. Moreover, we calculated a between-group Cohen’s d effect size and the 95% confidence interval. We interpreted the effect size as trivial (< 0.2), small (> 0.2), medium (> 0.5), large (> 0.8), and very large (> 1.3) [35, 36].

The percentages of missing values were 3.7 to 7.3% for HRV outcomes and 1.2 to 3.7% for step counts, with 93 and 96% of the 82 participants being included in the analysis using the traditional listwise deletion method. Data were missing primarily due to participant attrition, while item nonresponse was also observed. The multiple imputation (MI) technique was employed to address the problem of missing data under the assumption that missing values were missing at random [37]. Stata 15’s ‘mi impute’ command generated 20 imputed datasets, and visual inspection of imputation convergence led to 0 burn-in iterations [38]. Analyses conducted with each dataset were pooled according to Rubin’s rules [39]. Imputed values were comparable to observed values [see Additional file 1: Table S1]. In addition, the last-observation-carried-forward (LOCF) method was also used as another alternative to handle data missing at the follow-up. The results using LOCF were similar to MI [see Additional file 1: Tables S2, S3 and S4]; therefore, we presented MI imputed results. STATA version 15 (Stata Corp. 2017. Stata Statistical Software: Release 15. College Station, TX: StataCorp LLC) was used in all statistical analyses.

## Results

### Participants characteristics

Table 1 summarizes the demographics and baseline characteristics of both study groups The BA and usual care participants did not significantly differ in age, gender, marital status, monthly income, living status, and disease history. In both groups, the MMSE scores were also similar. Baseline TGDS, number of steps, and HRV: SDNN, LF, lnHF, and LF/HF were not significantly different. However, the number of those with no education was significantly lower, while the number of unemployed was significantly higher in the BA group.

**Table 1:** Demographic Characteristics of Study Participants (*n* = 82)

Variable	BA with usual care group (*n* = 41)	Usual care only group (*n* = 41)
Age, years: mean (SD)	70.54 (5.39)	69.20 (7.06)
Gender, Male: female: n (%)	8 (19.51): 33 (80.49)	9 (21.95): 32 (78.05)
Marital status: n (%)		
Single	6 (14.63)	8 (19.51)
Married	22 (53.66)	23 (56.10)
Widowed/Divorced/Separated	13 (31.71)	10 (24.39)
Education, n (%)		
None	2 (4.88)	12 (29.27)
Primary school	38 (92.68)	25 (60.97)
Secondary school and above	1 (2.44)	4 (9.76)
Employment, n (%)		
Unemployed	13 (31.70)	10 (24.39)
Agriculture	5 (12.20)	6 (14.63)
Merchant	8 (19.51)	5 (12.20)
Self employed	10 (24.39)	16 (39.02)
Housewife/husband	5 (12.20)	4 (9.76)
Living situation, n (%)		
Living alone	5 (12.20)	3 (7.32)
Live with spouse and children	29 (70.73)	27 (65.85)
Live with relatives	7 (17.07)	11 (26.83)
Income: Median (IQR)	800 (1,300)	700 (2,400)
Underlying diseases, (No: Yes): n (%)	7 (17.07): 34 (82.93)	6 (14.63): 35 (85.37)
MMSE score: mean (SD)	19.12 (1.72)	18.95 (2.37)
TGDS: mean (SD)	16.49 (2.73)	17.07 (2.68)
Walking steps per day: Median (IQR)	1,327.14 (2,643.86)	1,310.80 (2,328.06)
Heart Rate Variability (HRV)		
SDNN (Ms)	23.10 (12.66)	24.07 (11.28)
lnHF (ms2)	4.81 (0.89)	4.85 (0.96)
LF (ms2)	5.04 (1.09)	5.06 (0.99)
LF/HF (ms2)	1.05 (0.18)	1.06 (0.18)

Abbreviations: *MMSE* mental state examination, *SD* standard deviation, *lnHF* high frequency, *LF* low frequency, *LF/HF* Low/high frequency ratio, *SDNN* standard deviation of the NN interval

### Effects of BA on daily step counts

**Fig. 2:**
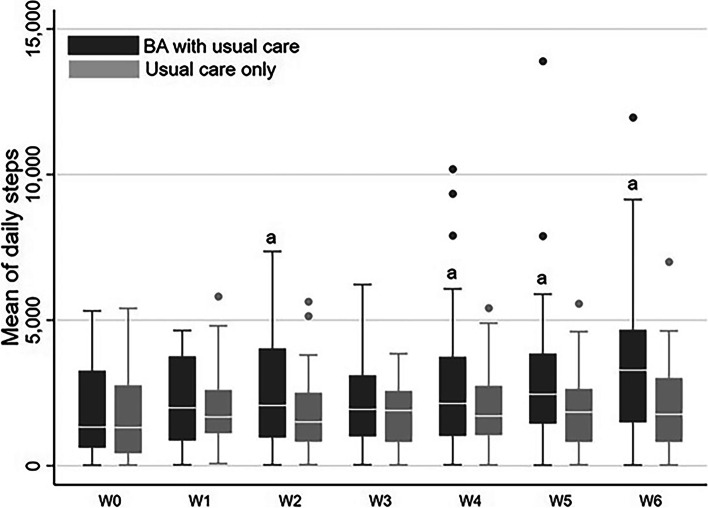
Mean boxplot of daily steps at each time point of the two study groups. Abbreviations: BA, behavioral activation; W, week; a, significant differences within BA with usual care

Comparing the daily steps data across time points within the BA and the usual care groups indicated that the number of daily steps at W2, W4, W5, and W6 increased significantly compared to baseline, with no significant within-group differences in daily steps in the usual care only group [see Additional file 1: Tables S3 and S4]. Furthermore, no significant between-group differences in daily steps were observed (Table 2, Fig. 2).

**Table 2:** Comparison of the Numbers of Daily Steps for the Two Study Groups

Outcome measures	GEEs (Mean ± SD)						
	**W0**	**W1**	**W2**	**W3**	**W4**	**W5**	**W6**
BA with usual care group	1,896.75 ± 1,664,96	2,264.61 ± 1,503.09	2,584.41 ± 2,108.21	2,324.25 ± 1,736.62	2,737.96 ± 2,367.04	3,076.55 ± 2,462.77	3,382.94 ± 2,320.26
Usual care only group	1,716.29 ± 1,441.25	1,872.95 ± 1,309.68	1,775.39 ± 1,358.83	1,785.22 ± 1,153.96	1,974.60 ± 1,417,94	1,974.60 ± 1,417,94	2,032.91 ± 1,502.62

Abbreviations: *BA* behavioral activation, *GEEs* generalized estimating equations, *W* week

### Effect of BA on HRV outcomes

**Fig. 3:**
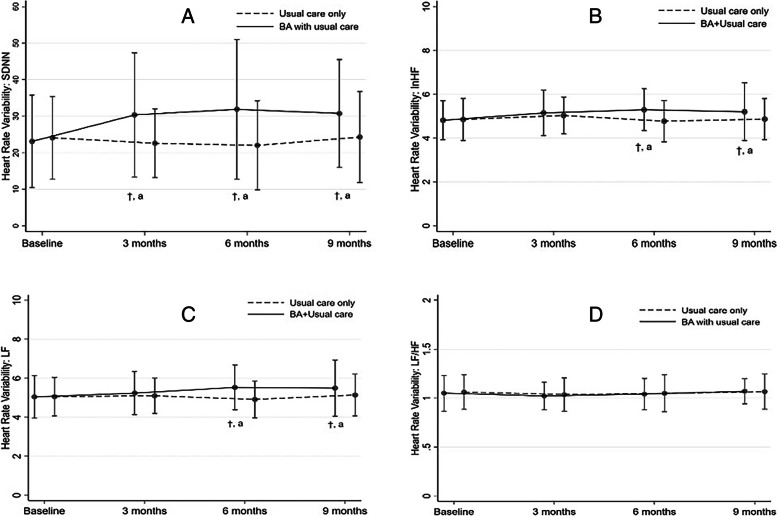
Mean and standard deviation of heart rate variability line charts at each time point. **A** SDNN (ms) standard deviation of the normal-to-normal interval, (**B)** InHF (ms^2^) high frequency, (**C)** LF (ms^2^) low frequency, (**D)** LF/HF (ms^2^) low frequency/high frequency ratio. Abbreviations: BA, behavioral activation; t, *p* < 0.05 between groups; a, significantly differ from T0 for within-group comparison

SDNN significantly improved from 3 to 9 months in the BA with the usual care group compared to the baseline (Table 3, Fig. 3 (A, B)). lnHF and LF significantly improved only from 6 to 9 months (Table 3, Fig. 3 (C)), while such improvement was not observed for LF/HF ratio (Table 3, Fig. 3 (D)). No significant change in HRV outcomes was observed in the usual care-only group during the follow-up period (Table 3, Fig. 3).

**Table 3:** Results of the Generalized Estimating Equation Model of HRV and Cohen’s d Effect Sizes

	**Mean** ± **SD**		**Mean Difference (95% CI)**		**Cohen’s d Effect Size (95% CI)**^**b**^
	**BA with usual care group**	**Usual care only group**	**Unadjusted**	**Adjusted**^**a**^	
**SDNN (ms)**					
Baseline	23.10 ± 12.66	24.07 ± 11.28	-0.98 (-6.89, 4.94)	-0.48 (-6.69, 5.73)	
3 months	29.85 ± 17.76	22.58 ± 9.27	7.26 (1.34, 13.18)*	7.75 (1.54, 13.96)*	-0.54 (-0.96, -0.09)
6 months	31.85 ± 18.89	22.09 ± 11.96	9.76 (3.84, 15.68)*	10.25 (4.04, 16.46)*	-0.62 (-1.06, -0.17)
9 months	31.83 ± 15.16	24.25 ± 12.16	7.59 (1.67, 13.50)*	8.08 (1.87, 14.29)*	-0.55 (-0.99, -0.11)
**lnHF (ms**^**2**^**)**					
Baseline	4.81 ± 0.89	4.85 ± 0.96	-0.04 (-0.44, 0.37)	0.10 (-0.32, 0.52)	
3 months	5.08 ± 1.06	5.02 ± 0.83	0.05 (-0.36, 0.45)	0.18 (-0.23, 0.60)	-0.05 (-0.48, 0.38)
6 months	5.26 ± 0.95	4.77 ± 0.92	0.49 (0.08, 0.90)*	0.63 (0.21, 1.05)*	-0.53 (-0.96, -0.08)
9 months	5.32 ± 1.08	4.88 ± 0.92	0.44 (0.04, 0.85)*	0.58 (0.16, 1.00)*	-0.44 (-0.88, -0.00)
**LF (ms**^**2**^**)**					
Baseline	5.04 ± 1.09	5.06 ± 0.99	-0.01 (-0.46, 0.43)	0.13 (-0.33, 0.59)	
3 months	5.19 ± 1.10	5.11 ± 0.89	0.08 (-0.37, 0.52)	0.22 (-0.24, 0.68)	-0.08 (-0.51, 0.36)
6 months	5.52 ± 1.12	4.86 ± 0.96	0.65 (0.20, 1.09)*	0.79 (0.33, 1.25)*	-0.62 (-1.06, -0.17)
9 months	5.67 ± 1.13	5.13 ± 1.04	0.53 (0.09, 0.98)*	0.67 (0.21, 1.13)*	-0.49 (-0.93, -0.05)
**LF/HF (ms**^**2**^**)**					
Baseline	1.05 ± 0.18	1.06 ± 0.18	0.01^c^ (-0.04, 0.06)	-0.00^C^ (-0.04, 0.05)	
3 months	1.03 ± 0.15	1.03 ± 0.17	0.01^c^ (-0.04, 0.06)	-0.00^C^ (-0.04, 0.05)	0.03 (-0.40, 0.46)
6 months	1.05 ± 0.16	1.03 ± 0.18	0.01^c^ (-0.04, 0.06)	-0.00^C^ (-0.04, 0.05)	-0.06 (-0.49, 0.38)
9 months	1.09 ± 0.14	1.06 ± 0.17	0.01^c^ (-0.04, 0.06)	-0.00^C^ (-0.04, 0.05)	-0.19 (-0.62, 0.25

^*^p < 0.05

^a^ GEEs was used to analyze the mean difference adjusted for employment status and education level of each outcome

^b^ Cohen’s d effect size at 3, 6, and 9 months compared to baseline interpreted as follows: trivial (< .2), small (> .2), medium (> .5), large (> .8), and very large (> 1.3)

^c^ no interaction effect changes over time

Abbreviations: *BA* behaviora l activation, *lnHF* high frequency, *LF* low frequency, *LF/HF* Low/high Frequency ratio, *SDNN* standard deviation of the NN interval, *95% CI* 95% confidence interval

The between-group comparison showed that the SDNN, lnHF, and LF improvements from 6 to 9 months follow-up were significantly higher in the BA with usual care group than in the usual care only group, with small to medium effect sizes (Cohen’s d values of 0.44–0.62) (Table 3, Fig. 3 (A, B)). On the other hand, the BA with the usual care group did not differ significantly from the usual care only group in the LF/HF ratio trend (the effect sizes were trivial; Cohen’s d values of 0.03–0.19, see Table 3, Fig. 3 C and D).

## Discussion

In this study, the older Thai participants with subthreshold depression showed a significant improvement in objective measures, such as the heart rate variability time domain of SDNN, frequency domain of lnHF band power and LF band power, following BA compared to the control group. Nevertheless, the improvements in LF/HF frequency ratio were not significant. Furthermore, BA significantly increased the participants' physical activity level during the intervention, as measured by daily step counts, although differences between the BA with the usual care group and the usual care only group were non-significant.

Our results demonstrated that BA induced significant changes in HRV by increasing the time domain of SDNN and frequency domain of lnHF and LF while decreasing the frequency domain of the LF/HF ratio. The HRV changes after BA indirectly indicate increased parasympathetic (vagal tone) and decreased sympathetic power. The BA group showed greater improvements in HRV compared to the usual care-only group. The parasympathetic tone also increased according to changes in SDNN, lnHF, and LF. The LF/HF ratio, as an index of sympathetic tone, did not change significantly. The increase in the sympathetic tone and decrease no change in HRV may indicate major depression [15, 40–42]. However, an increased amount of HRV can stimulate the parasympathetic tone. Thus, the parameters of HRV that increased in response to BA reflect well-regulated autonomic nervous system tone.

Many studies have elucidated that low HRV indicates the presence of symptoms or abnormal physical or mental health. Compared to healthy control groups, people with depression had lower SDNN, lnHF, and LF [15, 40–42]. Several studies have found a high LF/HF ratio in persons with depression [43, 44]. Recent research, however, has cast doubt on the LF/HF ratio's interpretation [45]. Autonomic imbalances in depression appear to be associated with altered cortical and subcortical networks functioning, most notably in the prefrontal cortex and the amygdala [15] In contrast, high HRV is linked to good health conditions and the ability to recover and perform various functions well [46] It can be measured by the amplitude of oscillations of HRV in both the time and frequency domains.

This report, therefore, could serve two scientific purposes. First, it strengthens our previously reported evidence of the BA effect on the clinically significant improvements in subjective outcomes, such as depression, stress, and symptoms, among Thai older participants with mild to moderate depression [25] Since these symptoms were assessed using the structured questionnaires, they were thus prone to response bias and the over-reporting of the beneficial effect of BA. The accompanying evidence of the BA effect on the improvement in physical activity and HRV levels measured using objective tools, such as pedometer and EKG, strongly supports the therapeutic or remedial effect of BA on the mental health of high-risk older adults. Although the effect sizes were small to medium, BA intervention can significantly influence public health, and it can be easily implemented with high-risk target populations because of its simplicity. Second, it provided the information that may elucidate or explain the mechanism through which BA improves mental health symptoms. The previous paragraph mentions evidence supporting the biologically plausible link between HRV level and mental health symptoms. Therefore, our results about the effect of BA on HRV improvement imply that BA exerts therapeutic or remedial influence on depression, stress, and anxiety via its effect on HRV improvement.

Our post hoc findings of significant inverse correlation between the HRV parameters (SDNN, lnHF and LF) and mental health symptom scores (the Thai geriatric depression scale or TGDS and Depression Anxiety Stress Scales or DASS; see Additional file 1: Table S6) were consistent with this presumption. However, as the correlations were low (Pearson correlations ranged from -0.12 to -0.21, see Additional file 1: Table S6), further studies are needed.

Two possible explanations could clarify the potential mechanism through which BA affects HRV improvement. First, BA may affect HRV via behavior changes, particularly the increased physical activity level. Our observation showed that BA participants significantly increased their daily steps during the BA intervention compared to baseline. Post hoc analysis also showed that daily steps significantly and positively correlated with HRV parameters (SDNN, lnHF and LF), although the correlation magnitudes were rather low (Pearson correlations ranged from 0.11 to 0.17, Additional file 1: Table S6). This was supported by the existing evidence showing that physical activity can improve HRV in older adults [47, 48] In addition, previous studies have shown that mind–body exercise (Tai Chi) and the combination of structured physical exercise with an antidepressant (sertraline) might positively affect the autonomic control of the heart among older patients with major depression [49, 50]. However, more evidence is needed since our BA participants did not significantly differ from the control group participants in the increase in daily steps.

Second, changes in thoughts and attitudes induced by the activities completed throughout the BA course may have further changed the HRV level. This was supported by the bidirectional connection between the parasympathetic nervous system and the prefrontal cortex area involved in emotion regulation and behavior control [51]. Additionally, an imaging study demonstrated the association between HRV and activity of the prefrontal cortex in the context of emotion regulation [17]. This study also revealed an association between higher HRV and stronger functional connectivity between the amygdala and the prefrontal cortex [52], a shape associated with emotion regulation in both younger and older adults [53]. In addition, a previous meta-analysis indicated an association between emotion regulation and HRV via the common brain regions involved in both systems [54]. Specifically, HRV was significantly associated with regional cerebral blood flow in the ventromedial prefrontal cortex and the amygdala [54]. Additionally, greater structural thickness in prefrontal regions was associated with greater HRV in younger and older adults [53].

Compared to other techniques used to improve HRV, our reported medium effect size for time-domain measures of SDNN interval and trivial to small effect size for frequency domain measures LF, lnHF, and LF/HF ratio were consistent with a recent systematic review and meta-analysis of mindfulness by Rådmark and et al. [55]. Additionally, a recent meta-analysis of mind–body interventions (Yoga and Tai-Chi) by Zou et al. found small to moderate beneficial effects on HRV of LF, lnHF and LF/HF [56]. Previous literature on the effects of mindfulness on HRV proved that increased SDNN, RMSDD, and lnHF were associated with better parasympathetic function and well-being [57]. Mind–body exercises (Tai Chi) [50] help increase HRV or modulate the autonomous nervous system.

### Study strengths and limitations

This was the first study to demonstrate that BA improved various HRV indices in older adults with subthreshold depression. Most participants' treatment compliance was high (92.7%), resulting in an adequate statistical power to detect the relationships proposed in this study. A non-mental health professional who delivered the BA showed the potential for extensive public health application in environments with limited resources. Nevertheless, the study had some limitations. First, the assessment of HRV could change based on various factors, such as age, gender, mental state, smoking, drinking, exercise, disease, and others. However, we minimized the interference of these factors by meticulous participant recruitment and disclosure of initial preparations before each HRV exam session. Second, the assessment of HRV using ultra- short-term (less than 5 min) HRV analyses remained a limitation because it should be performed with a large population consisting of different age groups, and the data should be collected in well-defined, controlled environments. Third, stationarity violation and artifact contamination were not corrected during the HRV assessment. Last, as the study period was only nine months, whether the BA effect on HRV is maintained over the long term is unknown.

## Conclusion

The current study supports efficacy of BA in improving HRV, including SDNN, lnHF parasympathetic nervous system, and LF sympathetic nervous system, in older Thai adults with subthreshold depression.

### Clinical Implications

BA may have a therapeutic effect on depression symptoms of older adults with subthreshold depression via improving their HRV and stimulating the parasympathetic tone. Thus, the parameters of HRV that increased in response to BA reflect well-regulated autonomic nervous system tone. Moreover, physical activity could be indirectly linked and correlated with improved depressive symptoms and HRV indices.

## Supplementary Information

**Additional file 1: Table S1**. Summary measures of heart rate variability parameters and daily steps of the original versus the imputed datasets.** Table S2. **Comparison of the Numbers of Daily Steps for the Two Study Groups, LOCF method. **Table S3. **Comparison of the numbers of daily steps within groups (Generalized Mixed Model) LOCF method†. **Table S4. **Comparison of the numbers of daily steps within groups (Generalized Mixed Model), MI method†. **Table S5. ** Results of the Generalized Estimating Equation Model of HRV and Cohen’s d Effect Sizes**, **LOCF method†. **Table S6.** Pearson’s correlation coefficient values showing correlations among daily steps, heart rate variability parameters, and depression, anxiety, and stress scores.

## Data Availability

The datasets used and/or analysed during the current study available from the corresponding author on reasonable request.

## Abbreviations

BA: Behavioral activation
CBT: Cognitive Behavior Therapy
CI: Confidence Interval
DASS: Depression Anxiety Stress Scales
ECG: Electrocardiogram
GEEs: Generalized Estimating Equations
lnHF: High frequency
HPH: Health Promoting Hospitals
HRV: Heart rate variability
IQR: Interquartile range
ITT: Intention-to-treat
LF: Low frequency
LF/HF: Low/high Frequency ratio
LOCF: Last-observation-carried-forward
MMSE: Mental State Examination
M: Month
PSD: Power spectral density
RCT: Randomized control trial
SD: Standard deviation
SDNN: Standard Deviation of the Normal-to-Normal interval
TGDS: Thai Geriatric Gepression Scale
T0: Baseline
T1: At the end of BA intervention of the experimental group or at the end of the 3rd month of the study
T2: 3 Months follow-up after the end of the intervention or at the end of the 6th month of the study
T3: 6 Months follow-up after the end of the intervention or at the end of the 9th month of the study
VHVs: Village health volunteers
W: Week
