# Supplementary material for: Effect of behavioral activation on time and frequency domain heart rate variability in older adults with subthreshold depression: a cluster randomized controlled trial in Thailand
Source: BMC Psychiatry. 2022 May 5;22:319. doi: 10.1186/s12888-022-03962-8 (PMC9069783; doi:10.1186/s12888-022-03962-8)
Supplement: Supplementary file 1 — Additional file 1: Table S1. Summary measures of heart rate variability parameters and daily steps of the original versus the imputed datasets. Table S2. Comparison of the Numbers of Daily Steps for the Two Study Groups, LOCF method. Table S3. Comparison of the numbers of daily steps within groups (Generalized Mixed Model) LOCF method†. Table S4. Comparison of the numbers of daily steps within groups (Generalized Mixed Model), MI method†. Table S5. Results of the Generalized Estimating Equation Model of HRV and Cohen’s d Effect Sizes, LOCF method†. Table S6. Pearson’s correlation coefficient values showing correlations among daily steps, heart rate variability parameters, and depression, anxiety, and stress scores. [file 12888_2022_3962_MOESM1_ESM.docx]

**Additional file 1**

**Table S1** Summary measures of heart rate variability parameters and daily steps of the original versus
 the imputed datasets

| **Variables** | **# Missing (n)** | **Original Dataset (Mean ± SD)** | |  | **Imputed Dataset† (Mean ± SD)** | |
| --- | --- | --- | --- | --- | --- | --- |
|  |  | **BA with usual care group (n=37)** | **Usual care only group (n=39)** |  | **BA with usual care group (n=41)** | **Usual care only group (n=41)** |
| **SDNN** |  |  |  |  |  |  |
| Baseline | - | 23.40 ± 12.90 | 23.92 ± 11.38 |  | 23.10 ± 12.66 | 24.07 ± 11.28 |
| 3 months | 3 | 30.34 ± 17.01 | 22.59 ± 9.38 |  | 29.85 ± 17.76 | 22.58 ± 9.27 |
| 6 months | 4 | 31.06 ± 14.04 | 22.02 ± 12.19 |  | 31.85 ± 18.89 | 22.09 ± 11.96 |
| 9 months | 6 | 31.58 ± 14.04 | 24.27 ± 12.44 |  | 31.83 ± 15.16 | 24.25 ± 12.16 |
| **lnHF (ms^2^)** |  |  |  |  |  |  |
| Baseline | - | 4.86 ± 0.89 | 4.86 ± 0.97 |  | 4.81 ± 0.89 | 4.85 ± 0.96 |
| 3 months | 3 | 5.15 ± 1.04 | 5.03 ± 0.84 |  | 5.08 ± 1.06 | 5.02 ± 0.83 |
| 6 months | 4 | 5.25 ± 0.91 | 4.77 ± 0.94 |  | 5.26 ± 0.95 | 4.77 ± 0.92 |
| 9 months | 6 | 5.34 ± 1.01 | 4..87 ± 0.93 |  | 5.32 ± 1.08 | 4.88 ± 0.92 |
| **LF (ms^2^)** |  |  |  |  |  |  |
| Baseline | - | 5.10 ± 1.08 | 5.03 ± 0.99 |  | 5.04 ± 1.09 | 5.06 ± 0.99 |
| 3 months | 3 | 5.24 ± 1.10 | 5.10 ± 0.90 |  | 5.19 ± 1.10 | 5.11 ± 0.89 |
| 6 months | 4 | 5.48 ± 1.13 | 4.92 ± 0.94 |  | 5.52 ± 1.12 | 4.86 ± 0.96 |
| 9 months | 6 | 5.64 ± 1.13 | 5.14 ± 1.07 |  | 5.67 ± 1.13 | 5.13 ± 1.04 |
| **LF/HF (ms2)** |  |  |  |  |  |  |
| Baseline | - | 1.05 ± 0.19 | 1.06 ± 0.17 |  | 1.05 ± 0.18 | 1.06 ± 0.18 |
| 3 months | 3 | 1.02 ± 0.14 | 1.03 ± 0.17 |  | 1.03 ± 0.15 | 1.03 ± 0.17 |
| 6 months | 4 | 1.05 ± 0.17 | 1.05 ± 0.19 |  | 1.05 ± 0.16 | 1.03 ± 0.18 |
| 9 months | 6 | 1.08 ± 0.13 | 1.06 ± 0.18 |  | 1.09 ± 0.14 | 1.06 ± 0.17 |
| **Daily Steps** |  |  |  |  |  |  |
| Baseline | - | 1,896.75 ± 1,664.96 | 1,716.29 ± 1,441.25 |  | 1,896.75 ± 1,664,96 | 1,716.29 ±1,441.25 |
| 1 weeks | - | 2,264.61 ± 1,503.09 | 1,872.95 ± 1,309.68 |  | 2,264.61 ± 1,503.09 | 1,872.95 ± 1,309.68 |
| 2 weeks | 1 | 2,584.41 ± 2,108.21 | 1,806.02 ± 1,321.69 |  | 2,584.41 ± 2,108.21 | 1,775.39 ± 1,358.83 |
| 3 weeks | 1 | 2,324,25 ± 1,736.62 | 1,825.55 ± 1,139.02 |  | 2,324.25 ± 1,736.62 | 1,785.22 ± 1,153.96 |
| 4 weeks | 2 | 2,740.43 ± 2,397.14 | 1,995.09 ± 1,429.85 |  | 2,737.96 ± 2,367.04 | 1,974.60 ± 1,417,94 |
| 5 weeks | 2 | 3,086.81 ± 2,493.26 | 1,960.12 ± 1,355.38 |  | 3,076.55 ± 2,462.77 | 1,951.39 ± 1,339.50 |
| 6 weeks | 3 | 3,483.58 ± 2,550.09 | 2,055.51 ± 1,514.69 |  | 3,382.94 ± 2,320.26 | 2,032.91 ± 1,502.62 |

**Abbreviations:** BA, behavioral activation; lnHF, high frequency; LF, low frequency; LF/HF, Low/high Frequency ratio; SDNN, standard deviation of the NN interval; 95% CI, 95% confidence interval

†Multiple imputation (MI) method was used to handle missing data

**Table S2**  Comparison of the Numbers of Daily Steps for the Two Study Groups, LOCF method†

| **Outcome measures** | **GEE Adjusted (Mean ± SD)** | | | | | | |
| --- | --- | --- | --- | --- | --- | --- | --- |
|  | **W0** | **W1** | **W2** | **W3** | **W4** | **W5** | **W6** |
| BA with usual care group | 1,327.14 ±2,643.86 | 1,986.14 ±2,883.04 | 2,068.86 ±3,061.00 | 1,936.80 ±2,091.12 | 2,139.43 ±2,713.14 | 2,453.71 ±2,409.00 | 3,281.71 ±3,178.40 |
| Usual care only group | 1,310.80 ±2,328.06 | 1,674.70 ±1,478.30 | 1,503.57 ±1,677.57 | 1,898.00 ±1,747.14 | 1,707.29 ±1,683.10 | 1,842.14 ±1,816.14 | 1,766.71 ±2,184.57 |

**Abbreviations:** BA, behavioral activation; GEE, generalized estimating equation; W, Week

†the last-observation-carried-forward (LOCF) method was used to handle missing data

**Table S3** Comparison of the numbers of daily steps within groups (Generalized Mixed Model)

LOCF method†

| **Outcome measures** | **Adjusted Mean Difference (95% CI) ^a^** | |
| --- | --- | --- |
|  | **BA with usual care group** | **Usual care only group** |
| Week 1 – Week 0 | 367.86 (-65.30, 801.02) | 156.66 (-276.50, 589.82) |
| Week 2 – Week 0 | 687.66 (237.63, 1,137.68)* | 50.35 (-399.67, 500.37) |
| Week 3 – Week 0 | 427.49 (-49.31, 904.29) | 69.39 (-407.41, 546.20) |
| Week 4 – Week 0 | 801.93 (289.99, 1,313.88)* | 234.81 (-277.14, 746.75) |
| Week 5 – Week 0 | 1,139.87 (586.00, 1,693.73)** | 200.69 (-353.18, 754.55) |
| Week 6 – Week 0 | 1,485.58 (884.43, 2,086.72)** | 293.75 (-307.39, 894.89) |

**p-value < 0.001, *****p-value < 0.05

**Note:** ^a^GEE was used to analyze the mean difference adjusted for employment status and education level of each outcome

†the last-observation-carried-forward (LOCF) method was used to handle missing data

**Table S4** Comparison of the numbers of daily steps within groups (Generalized Mixed Model),

MI method†

| **Outcome measures** | **Adjusted Mean Difference (95% CI)** | |
| --- | --- | --- |
|  | **BA with usual care group** | **Usual care only group** |
| Week 1 – Week 0 | 367.86 (-66.70, 802.43) | 156.66 (-277.91, 591.22) |
| Week 2 – Week 0 | 687.66 (235.87, 1,139.44)* | 59.10 (-392.69, 510.88) |
| Week 3 – Week 0 | 427.49 (-51.62, 906.61) | 68.92 (-410.19, 548.04) |
| Week 4 – Week 0 | 841.20 (326.26, 1,356.15)* | 258.31 (-256.64, 773.26) |
| Week 5 – Week 0 | 1,179.80 (622.15, 1,737.44)** | 235.10 (-322.55, 792.74) |
| Week 6 – Week 0 | 1,486.19 (880.43, 2,091.94)** | 316.62 (-289.13, 922.37) |

**p-value < 0.001, *****p-value < 0.05

†Multiple imputation (MI) method was used to handle missing data

**Table S5**  Results of the Generalized Estimating Equation Model of HRV and Cohen’s d Effect Sizes**,**

LOCF method†

|  | **Mean** ± **SD** | | **Mean Difference (95% CI)** | | **Cohen’s d Effect Size (95% CI)^b^** |
| --- | --- | --- | --- | --- | --- |
|  | **BA with usual care group** | **Usual care only group** | **Unadjusted** | **Adjusted^a^** |  |
| **SDNN (ms)** | |  |  |  |  |
| Baseline | 23.10 ± 12.66 | 24.07 ± 11.28 | -0.96 (-6.94, 4.97) | -0.53 (-7.02, 5.96) |  |
| 3 months | 29.70 ± 16.83 | 22.78 ± 9.34 | 6.92 (0.96, 12.88)* | 7.36 (0.88, 13.85)* | -0.51 (-0.95, -0.07) |
| 6 months | 31.18 ± 18.95 | 21.94 ± 12.09 | 9.23 (3.27, 15.19)* | 9.67 (3.18, 16.16)* | -0.58 (-1.02, -0.14) |
| 9 months | 31.64 ± 15.48 | 24.08 ± 12.35 | 7.56 (1.59, 13.52)* | 8.00 (1.51, 14.49)* | -0.54 (-0.98, -0.10) |
| **lnHF (ms^2^)** |  |  |  |  |  |
| Baseline | 4.81 ± 0.89 | 4.85 ± 0.96 | -0.04 (-0.45, 0.37) | 0.11 (-0.32, 0.55) |  |
| 3 months | 5.09 ± 1.05 | 5.02 ± 0.83 | 0.07 (-0.34, 0.48) | 0.22 (-0.22, 0.65) | -0.07 (-0.50, 0.36) |
| 6 months | 5.23 ± 0.98 | 4.78 ± 0.92 | 0.45 (0.04, 0.85)* | 0.60 (0.16, 1.03)* | -0.47 (-0.91, -0.03) |
| 9 months | 5.31 ± 1.06 | 4.87 ± 0.92 | 0.44 (0.03, 0.85)* | 0.59 (0.16, 1.03)* | -0.44 (-0.88, -0.01) |
| **LF (ms^2^)** |  |  |  |  |  |
| Baseline | 5.04 ± 1.09 | 5.06 ± 0.99 | 0.24c (-0.12, 0.60) | 0.36c (-0.04, 0.77) |  |
| 3 months | 5.17 ± 1.11 | 5.12 ± 0.90 | 0.24c (-0.12, 0.60) | 0.36c (-0.04, 0.77) | -0.05 (-0.48, 0.38) |
| 6 months | 5.45 ± 1.17 | 4.94 ± 0.94 | 0.24c (-0.12, 0.60) | 0.36c (-0.04, 0.77) | -0.48 (-0.92, -0.04) |
| 9 months | 5.60 ± 1.19 | 5.16 ± 1.05 | 0.24c (-0.12, 0.60) | 0.36c (-0.04, 0.77) | -0.39 (-0.83, 0.05) |
| **LF/HF (ms^2^)** | |  |  |  |  |
| Baseline | 1.05 ± 0.18 | 1.06 ± 0.18 | -0.01c (-0.06, 0.04) | -0.02c (-0.08, 0.04) |  |
| 3 months | 1.02 ± 0.14 | 1.04 ± 0.17 | -0.01c (-0.06, 0.04) | -0.02c (-0.08, 0.04) | 0.09 (-0.34, 0.53) |
| 6 months | 1.04 ± 0.16 | 1.05 ± 0.19 | -0.01c (-0.06, 0.04) | -0.02c (-0.08, 0.04) | 0.04 (-0.39, 0.47) |
| 9 months | 1.07 ± 0.13 | 1.07 ± 0.18 | -0.01c (-0.06, 0.04) | -0.02c (-0.08, 0.04) | -0.03 (-0.46, 0.40 |

**Note:** *p < 0.05; ^a^ GEE was used to analyze the mean difference adjusted for employment status and education level of each outcome; ^b^ Cohen’s d effect size at 3, 6, and 9 months compared to baseline interpreted as follows: trivial (<.2), small (>.2), medium (>.5), large (>.8), and very large (> 1.3); ^c^no interaction effect changes over time.

**Abbreviations:** BA, behavioral activation; lnHF, high frequency; LF, low frequency; LF/HF, Low/high Frequency ratio; SDNN, standard deviation of the NN interval; 95% CI, 95% confidence interval

†the last-observation-carried-forward (LOCF) method was used as the other alternative to handle missing data

**Table S6** Pearson’s correlation coefficient values showing correlations among daily steps, heart rate
 variability parameters, and depression, anxiety, and stress scores

|  | **Depression, Anxiety and Stress Scores** | | | | **Heart Rate Variability Parameters** | | | | **Daily Steps** |
| --- | --- | --- | --- | --- | --- | --- | --- | --- | --- |
|  | **TGDS** | **DASS (Depression)** | **DASS (Anxiety)** | **DASS (Stress)** | **SDNN** | **lnHF** | **LF** | **LF/HF** |  |
| **TGDS** |  | 0.57** | 0.40** | 0.54** | -0.21** | -0.15* | -0.15* | -0.01 | -0.15* |
| **DASS (Depression)** |  |  | 0.30** | 0.50** | -0.07 | -0.03 | -0.02 | -0.03 | -0.12* |
| **DASS (Anxiety)** |  |  |  | 0.36** | -0.14* | -0.15* | -0.12* | 0.03 | -0.14* |
| **DASS (Stress)** |  |  |  |  | -0.14* | -0.14* | -0.08 | -0.06 | -0.11 |
| **SDNN** |  |  |  |  |  | 0.70** | 0.72** | 0.04 | 0.14* |
| **lnHF** |  |  |  |  |  |  | 0.73** | -0.34** | 0.17* |
| **LF** |  |  |  |  |  |  |  | 0.37** | 0.11* |
| **LF/HF** |  |  |  |  |  |  |  |  | -0.07 |

**p-value < 0.001, *****p-value < 0.05

**Abbreviations:** DASS, Depression Anxiety Stress Scales; lnHF, high frequency; LF, low frequency; LF/HF, Low/high Frequency ratio; SDNN, standard deviation of the NN interval; TGDS, Thai Geriatric Depression Scale;.95% CI, 95% confidence interval.
